# Symmetric data-driven fusion of diffusion tensor MRI: Age differences in white matter

**DOI:** 10.3389/fneur.2023.1094313

**Published:** 2023-04-17

**Authors:** Andrea Mendez Colmenares, Michelle B. Hefner, Vince D. Calhoun, Elizabeth A. Salerno, Jason Fanning, Neha P. Gothe, Edward McAuley, Arthur F. Kramer, Agnieszka Z. Burzynska

**Affiliations:** ^1^BRAiN Laboratory, Department of Human Development and Family Studies, Colorado State University, Fort Collins, CO, United States; ^2^Molecular, Cellular and Integrative Neurosciences, Colorado State University, Fort Collins, CO, United States; ^3^Tri-institutional Center for Translational Research in Neuroimaging and Data Science (TReNDS), Georgia State, Georgia Tech, Emory, Atlanta, GA, United States; ^4^Division of Public Health Sciences, Department of Surgery, Washington University School of Medicine in St. Louis, St. Louis, MO, United States; ^5^Department of Health and Exercise Sciences, Wake Forest University, Winston-Salem, NC, United States; ^6^Department of Kinesiology and Community Health, University of Illinois at Urbana-Champaign, Urbana, IL, United States; ^7^Beckman Institute for Advanced Science and Technology, University of Illinois at Urbana-Champaign, Urbana, IL, United States; ^8^Department of Psychology, Northeastern University, Boston, MA, United States; ^9^Department of Human Development and Family Studies, Colorado State University, Fort Collins, CO, United States

**Keywords:** aging, white matter, diffusion MRI, multimodal, fusion

## Abstract

In the past 20 years, white matter (WM) microstructure has been studied predominantly using diffusion tensor imaging (DTI). Decreases in fractional anisotropy (FA) and increases in mean (MD) and radial diffusivity (RD) have been consistently reported in healthy aging and neurodegenerative diseases. To date, DTI parameters have been studied individually (e.g., only FA) and separately (i.e., without using the joint information across them). This approach gives limited insights into WM pathology, increases the number of multiple comparisons, and yields inconsistent correlations with cognition. To take full advantage of the information in a DTI dataset, we present the first application of symmetric fusion to study healthy aging WM. This data-driven approach allows simultaneous examination of age differences in all four DTI parameters. We used multiset canonical correlation analysis with joint independent component analysis (mCCA + jICA) in cognitively healthy adults (age 20–33, *n* = 51 and age 60–79, *n* = 170). Four-way mCCA + jICA yielded one high-stability modality-shared component with co-variant patterns of age differences in RD and AD in the corpus callosum, internal capsule, and prefrontal WM. The mixing coefficients (or loading parameters) showed correlations with processing speed and fluid abilities that were not detected by unimodal analyses. In sum, mCCA + jICA allows data-driven identification of cognitively relevant multimodal components within the WM. The presented method should be further extended to clinical samples and other MR techniques (e.g., myelin water imaging) to test the potential of mCCA+jICA to discriminate between different WM disease etiologies and improve the diagnostic classification of WM diseases.

## Introduction

Degradation in myelin and axonal structure in the white matter (WM) is one of the fundamental mechanisms contributing to cognitive decline in normative aging and Alzheimer’s Disease and Related Dementias (1). However, *in vivo* age differences in WM microstructure mechanisms are only partially understood. This is because almost all neuroimaging studies on the WM microstructure in aging in the past 20 years have used diffusion MRI and, predominantly, diffusion tensor imaging (DTI) (2).

Fractional anisotropy (FA) is a measure of the directional dependence of diffusion (3) and is influenced by the fiber orientational coherence, fiber diameter, integrity, and density (4). Mean diffusivity (MD) reflects the total magnitude of diffusion within a voxel, which is inversely proportional to the density of physical obstructions, such as myelin and cellular membranes (4, 5). Radial diffusivity (RD) measures the magnitude of diffusion perpendicular to the primary orientation of WM tracts, which in WM is restricted by axonal and myelin membranes. Axial diffusivity (AD) is a measure of diffusion along the length of an axon and is thought to reflect chronic axonal injury. RD and AD have been linked to axonal damage and loss in myelin membrane integrity (6, 7). Notably, AD and RD are orthogonal, and FA and MD are mathematical combinations of AD and RD. However, it is important to remember that DTI measures are only proxies for WM microstructural integrity and are not specific to any underlying neurobiological mechanism (8). Decreased FA and increased MD, RD, and bidirectional differences in AD have been consistently reported in healthy aging and Alzheimer’s Disease and related dementias (9).

Importantly, most DTI studies on aging and dementia have used only a fraction of information available in a diffusion dataset. Typically, age differences have been reported either selectively (e.g., only FA), in arbitrarily selected regions (e.g., the corpus callosum), and separately (i.e., without using the joint information across them, for example, shared versus unique information across FA and RD). Therefore, the aim of this study was to evaluate the use of the joint information across all four DTI parameters to revisit age differences in the entire WM using a data-driven symmetric fusion analysis.

There are different types of multimodal analysis (10). At one end of the spectrum is the visual inspection of different data types. For example, the analysis of the spatial overlap of unimodal analyses. We have used this approach in our earlier work, attempting to delineate different microstructural mechanisms of WM aging from overlapping patterns of age differences in FA, MD, RD, and AD (11). However, the overlap of voxels showing significant differences in each parameter map does not measure the interaction among them. As a result, our interpretation of the patterns of WM aging remained inconclusive.

In the current study, we use data fusion on the opposite side of the spectrum, namely, symmetric data fusion, which treats multiple image types (or modalities) equally to take full advantage of their joint information (10, 12). We chose to use data-driven multiset canonical correlation analysis with joint independent component analysis (mCCA + jICA) (10, 13, 14). This method combines the flexibility of mCCA in maximizing covariations between the modalities (15) with superior source separation with jICA (14).

mCCA + jICA outputs modality-shared and modality-unique independent components (IC). These ICs represent sources of the signal, which—we hypothesize, based on unimodal analyses of DTI data—should be congruent with age-related processes in WM microstructure known from histological studies. For example, a modality-shared IC composed of decreased FA and increased MD, RD, and AD in older adults would likely reflect demyelination or chronic tissue loss (7, 11, 16). The retrogenesis hypothesis of brain aging (17) posits that WM regions that are last to myelinate during development are also most vulnerable to aging. Thus, we hypothesized that an IC reflecting demyelination or tissue loss would be localized predominantly to late-myelinating WM regions, such as the prefrontal WM, anterior corpus callosum, fornix, and the external capsule (18–20).

Next, with this data-driven, exploratory approach, we expected to obtain new insights into age differences in WM microstructure that cannot be identified with a single parameter map or image modality or by using traditional inferential statistics. Multimodal analyses using partial least squares (21) or linked ICA (22) showed great promise in identifying patterns of correlated group differences across diffusion MRI features to improve diagnostic classification between healthy controls and people at different stages of Alzheimer’s disease.

Finally, to date, unimodal analyses yielded mixed associations with cognition, with marked inconsistencies between WM regions or tracts, DTI parameters, and cognitive constructs, possibly hampered by the number of multiple comparisons (2, 23, 24). Therefore, we aimed to test whether multimodal fusion can identify components relevant to cognition. Specifically, we hypothesized that covariant DTI differences between young and old would be associated with executive functions and processing speed, the cognitive functions most affected by aging and possibly most sensitive to changes in brain’s structural connectivity *via* WM (25).

## Methods

### Participants

The MRI data used in this study were obtained from three studies conducted between 2011 and 2014 on neurologically and cognitively healthy adults. We acquired the data using the 3 T Siemens TIM Trio system with 45 mT/m gradients and 200 T/m/s slew rates (Siemens, Erlangen, Germany) at the Beckman Institute for Advanced Science and Technology at the University of Illinois, United States. All studies were approved by the University of Illinois at Urbana-Champaign Institutional Review Board, with written informed consent obtained from all participants.

#### Older adults

Data for older adults were obtained from the baseline MRI data of community-dwelling participants (*n* = 170), aged 60–79 years, in the Fit and Active Senior clinical trial (ID: NCT01472744). For more information, refer to Baniqued et al., Burzynska et al., Ehlers et al., Fanning et al., Mendez Colmenares et al., and Voss et al. (26–32).

#### Young adults

Data for young adults were collected in two separate studies. The first study included *n* = 37 female dancers (aged 18–33) and education-matched peers with no professional dance training, recruited from the student population at the University of Illinois (33). The second study comprised *n* = 14 college-age young adults, collected as a reference sample for the FAST clinical trial.

Our final sample consisted of 221 participants (*n* = 51 young and *n* = 170 older adults; see Supplementary material 1 for participant flow).

### Diffusion tensor imaging

Diffusion tensor imaging images were obtained with no interslice gap, with a twice-refocused spin echo single-shot Echo Planar Imaging sequence (34) to minimize eddy current-induced image distortions. The protocol consisted of a set of 30 non-collinear diffusion-weighted acquisitions with *b*-value = 1,000 s/mm^2^ and two T2-weighted *b*-value = 0 s/mm^2^ acquisitions, repeated two times, with 128 × 128 matrix, GRAPPA acceleration factor 2, flip angle = 90, and a bandwidth of 1,698 Hz/Px. The DTI acquisition for the young dancer sample differed slightly on voxel dimensions and field of view (TR/TE = 10,000/98 ms, 1.9 × 1.9 mm^2^ in-plane resolution, and 72 2-mm-thick slices for full brain coverage), from the other young and older samples (TR/TE = 5,500/98 ms, 1.7 × 1.7 mm^2^ in-plane resolution, and 40 3-mm-thick slices). DTI data were processed using the FSL Diffusion Toolbox v.3.0 (FDT: http://www.fmrib.ox.ac.uk/fsl) (31). We used the Tract-Based Spatial Statistics (TBSS) workflow (35) to align diffusion images into a 1 mm× 1 mm× 1 mm standard Montreal Neurological Institute (MNI152) space *via* the FMRIB58_FA template and project the center-of-tract values onto the WM skeleton. Our final sample consisted of 221 participants (*n* = 51 young and *n* = 170 older adults).

### Symmetric data fusion (mCCA + jICA)

**Figure 1:**
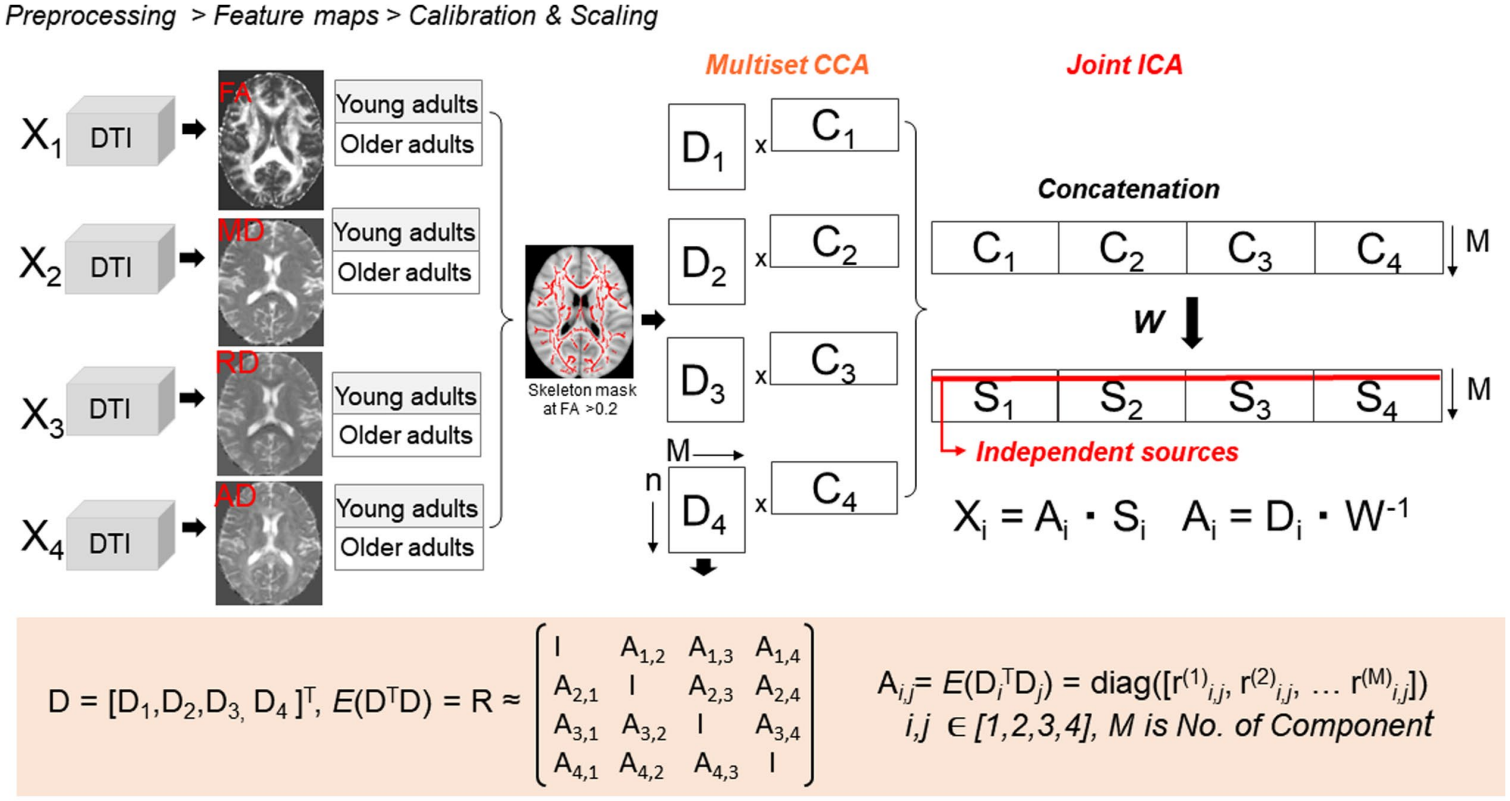
Four-way two-sample *t*-test mCCA + jICA. mCCA projects the data in a space so that the correlations among mixing profiles (D_k_, *k* = 1…*n*) of the four parameter maps are jointly maximized, resulting in canonical variates. Analyses were restricted to the WM using a TBSS-derived skeleton WM mask. D_k_ is then sorted by correlation to provide a closer initial match and make the further application of joint ICA more reliable. Joint ICA is then applied on the concatenated maps (C_n_) to obtain the final independent sources S_k_.

Multimodal age comparative analyses were carried out using a 4-way (FA, MD, RD, and AD) two-sample *t*-test mCCA + jICA (10, 13, 14, 36) using the Fusion ICA MATLAB Toolbox^1^ as described in Figure 1. We restricted our analyses to the WM skeleton thresholded at the default FA > 0.2.

### Model order

There are several ways of selecting the optimal model order (i.e., the number of resulting ICs), ranging from *a priori* to data-driven methods. Currently, there is no gold standard for selecting the model order for mCCA + jICA for exploring specifically skeletonized WM space. Therefore, to select our model order, we used *a priori* knowledge from postmortem histological examinations in humans and primates (37–43) as well as from spatial patterns of overlap in age differences in FA, MD, RD, and AD identified in earlier cross-sectional DTI studies (e.g., (11, 44)). The known histological age differences in WM include: (1) loss or thinning of myelin, (2) decrease in average axonal diameter, (3) loss of whole myelinated axons that may be associated with (4) decrease in tissue density and increase in extracellular (free) water or (5) increase in cellular density due to gliosis. Other histological changes in the aging WM include changes in axonal orientational alignment in a voxel due to (6) loss or rarefaction of fibers in a specific direction or (7) realignment due to macrostructural changes, as well as (8) changes in the microvasculature. Thus, we decided that a model with eight ICs would provide enough flexibility to accommodate a broad of possible microstructural processes yet be low enough to accommodate the restricted space of the WM skeleton (~8% of the total brain volume).

### IC quality assessment

We used 500 random iterations of ICA using the entropy-based minimization ICA (EBM ICA) algorithm (45). We used ICASSO to select the best single-run estimate to ensure the replicability of our results (46). ICASSO runs the ICA algorithm repeatedly and compares each result based on the correlation between squared source estimates (47). Next, ICASSO estimates the stability of the ICA using clustering analysis to compute a cluster quality index, Iq. We defined the Iq as {I = avg.[S(i)_int_]−avg[s(i)_ext_]}, where S is the spatial similarity between two ICs and i is the source matrix. Therefore, the Iq value represents the difference between intra- and inter-cluster component similarity. We used the quality index to assess the stability and reliability of the resulting ICs. Most studies use a quality index threshold between 80 and 90% (48–51); thus, we chose to examine only the ICs with an Iq > 0.90.

### mCCA + jICA

When applying the mCCA+jICA model, the 3D data were first reshaped to a one-dimensional vector by subject. Then, the data were normalized separately for each data type, ensuring that each data type has the same average sum of squares, which is computed across all subjects and voxels. This normalization process ensures that all features have the same ranges and contribute equally to the fusion model (52) (Figure 1). After running ICASSO, mCCA+jICA outputs a source matrix (loadings for each voxel) and a mixing matrix (loading coefficients for each component for each subject) (48). The mixing matrix allows for analyzing the inter-correlation between modalities and the differences between the groups (young vs. old). Therefore, modality-shared ICs (with significant mixing coefficients in at least two modalities) share variance across at least two feature maps, while modality-unique ICs represent unique variance. The mixing coefficients (also called loading parameters) reflect the degree to which a given component is expressed in each subject for a given feature. We used the GIFT Toolbox^2^ to plot the mixing coefficients in MATLAB. To visualize each independent component, each source matrix was reshaped to a 3D space, standardized (z-scored), and thresholded at *z* > 2.5 (*p* < 0.01, two-tailed). We tested the hypotheses by analyzing the composition, spatial location, and direction of age differences in the ICs. The composition of each IC is determined by the mixing coefficients and *p* values associated with its feature maps.

### Cognitive assessment

Cognitive assessment included the Virginia Cognitive Aging (VCAP) battery (53) administered as described in (32). Two cognitive composites were used in the analyses due to their reliance on WM integrity (2): executive function (matrix reasoning, Shipley abstraction, letter sets, spatial relations, paper folding, and form boards) and perceptual speed construct (digit symbol substitution, letter comparison, and pattern comparison). We calculated the composites as a sum of the z-score values across the respective tasks. Two subjects were missing data from all cognitive scores; these two subjects were included in the fusion analyses but not in the regression analyses with cognition. An additional five subjects were missing data for the “Letter Sets task” and two had missing data for the “Form Boards task” due to technical issues. For these seven subjects with missing data from one task, we replaced the missing score with the sample mean when calculating the composite scores, resulting in *n* = 219 for the final cognitive analyses.

### Statistics

The regression analysis between the mixing coefficients and cognition was corrected for family-wise error using the false discovery rate (FDR) method as implemented by *p*.adjust in R. We created figures using the ggplot function in the ggplot2 package (54). We performed statistical analyses in R version 4.2.1. Lastly, to minimize the effects of the outliers but to avoid removing data points, for both the mixing coefficients and the cognitive composites we identified outliers as <1st percentile or > 99th percentile of distribution (i.e., winsorized) by replacing them with the nearest value in the 1st or 99th percentile.

## Results

### Sample characteristics

The older and younger adults in our sample showed the expected age difference in speed and fluid abilities, as well as whole-skeleton DTI values, but did not differ in education. Additionally, the young adult group had a higher proportion of females than the older adult group (Table 1).

**Table 1:** Sample characteristics.

Variables	Young	Old	*p* value
	*n* = 51	*n* = 170	
Age	21.6 ± 3.2	65.4 ± 4.4	0.001
Women, *n* (%)	47 (91)	117 (68)	0.001
Education, years	15.4 ± 2.2	15.8 ± 2.9	0.409
DTI parameters			
FA	0.479 ± 0.02	0.454 ± 0.01	0.001
MD	0.753 ± 0.01	0.767 ± 0.03	0.001
RD	0.586 ± 0.09	0.507 ± 0.16	0.001
AD	0.661 ± 0.21	1.126 ± 0.09	0.001
Cognitive scores			
Digit symbol	82.96 ± 26.96	65.39 ± 13.79	0.001
Pattern comparison	19.05 ± 4.31	14.82 ± 2.57	0.001
Letter comparison	12.45 ± 2.94	9.53 ± 1.82	0.001
Letter sets	12.54 ± 2.09	11.05 ± 2.69	0.001
Spatial relations	12.05 ± 4.92	8.08 ± 4.73	0.001
Paper folding	8.57 ± 3.29	5.42 ± 2.57	0.001
Form boards	9.88 ± 4.41	5.60 ± 3.69	0.001
Shipley abstract	15.20 ± 2.58	12.36 ± 3.55	0.001
Matrix reasoning	11.49 ± 3.23	8.12 ± 3.03	0.001

MD, RD, and AD are expressed in μm^2^.ms^−1^. Values are presented as mean ± SD unless otherwise stated.

### mCCA + ICA output

**Figure 2:**
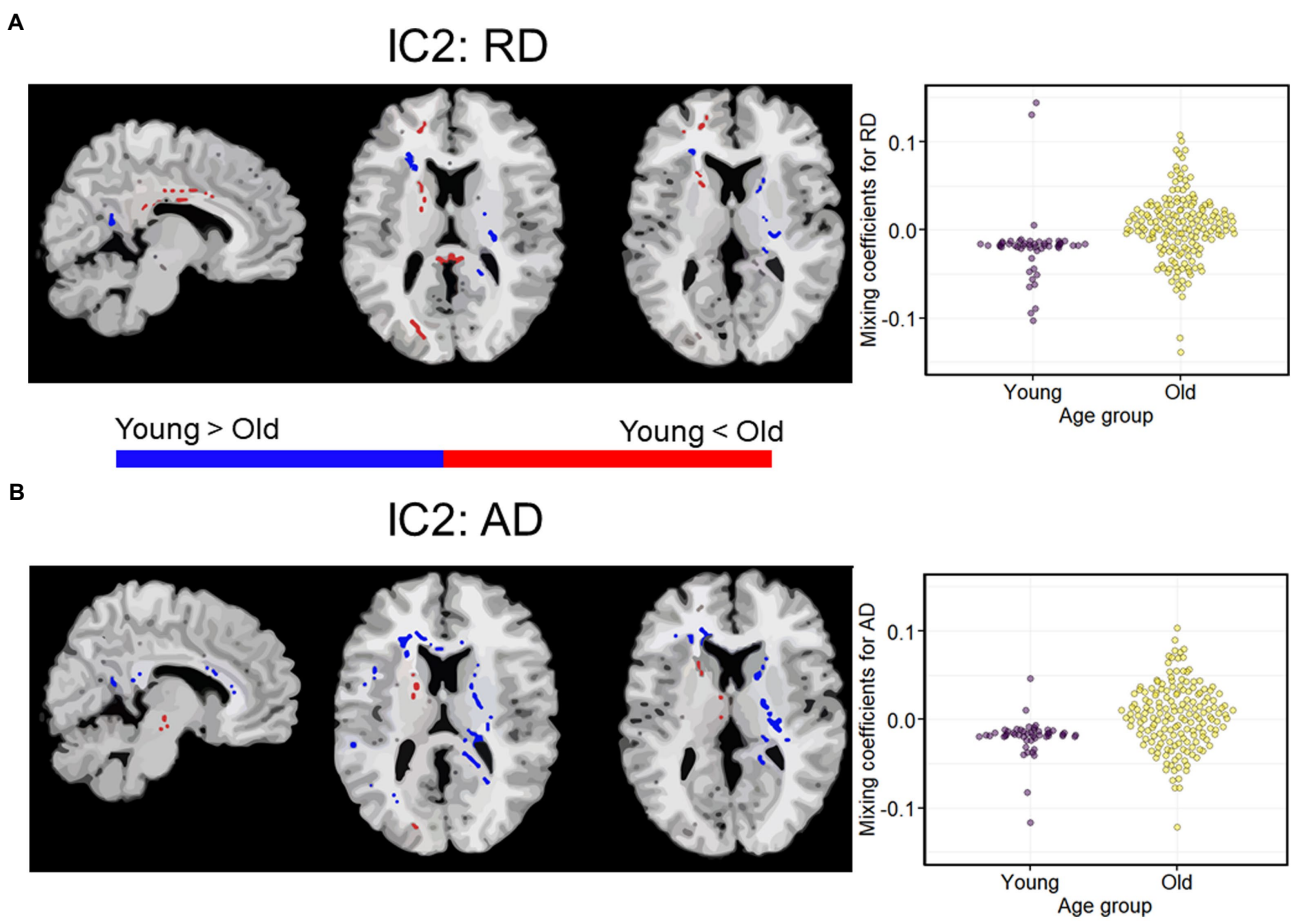
A modality-shared independent component (IC2) differentiating younger and older adults *via* independent samples t-test on mixing coefficients. **(A)** Spatial maps for RD. **(B)** Spatial maps for AD. When *z* scores (red voxels) are positive and mixing coefficients are positive, the component is showing increased RD/AD in older adults. Conversely, when *z*-scores are negative (blue voxels) and mixing coefficients are positive, the component is showing increased RD/AD in young adults. Density plots show the loading parameters (or mixing coefficients) of IC2 for both RD and AD feature maps. Higher mixing coefficients for both RD and AD in older adults means that IC2 was expressed more in older adults. All the two-sample *t*-tests between young and older adults had *p* < 0.01. IC, independent component.

Among the eight ICs, only one (IC2) had a qualifying Iq = 0.923. IC2 was a multimodal component with RD and AD showing significant age-discriminatory contributions. As shown in Figure 2, RD showed an increase in older adults in the right anterior and posterior internal capsule, body, and splenium of the corpus callosum, in the occipital WM, prefrontal WM, and frontal WM (anterior corona radiata and anterior cingulate; voxels in red). RD was decreased in older adults in fewer regions, which included the left anterior and posterior capsule, genu, and splenium corpus callosum (voxels in blue). AD was mostly decreased in older adults, which included the corpus callosum genu and splenium, right internal capsule, and prefrontal WM (blue). AD was increased in the older adults in a cluster of the left internal capsule and scattered voxels in the forceps minor and major (red).

### Mixing coefficients and cognition

**Figure 3:**
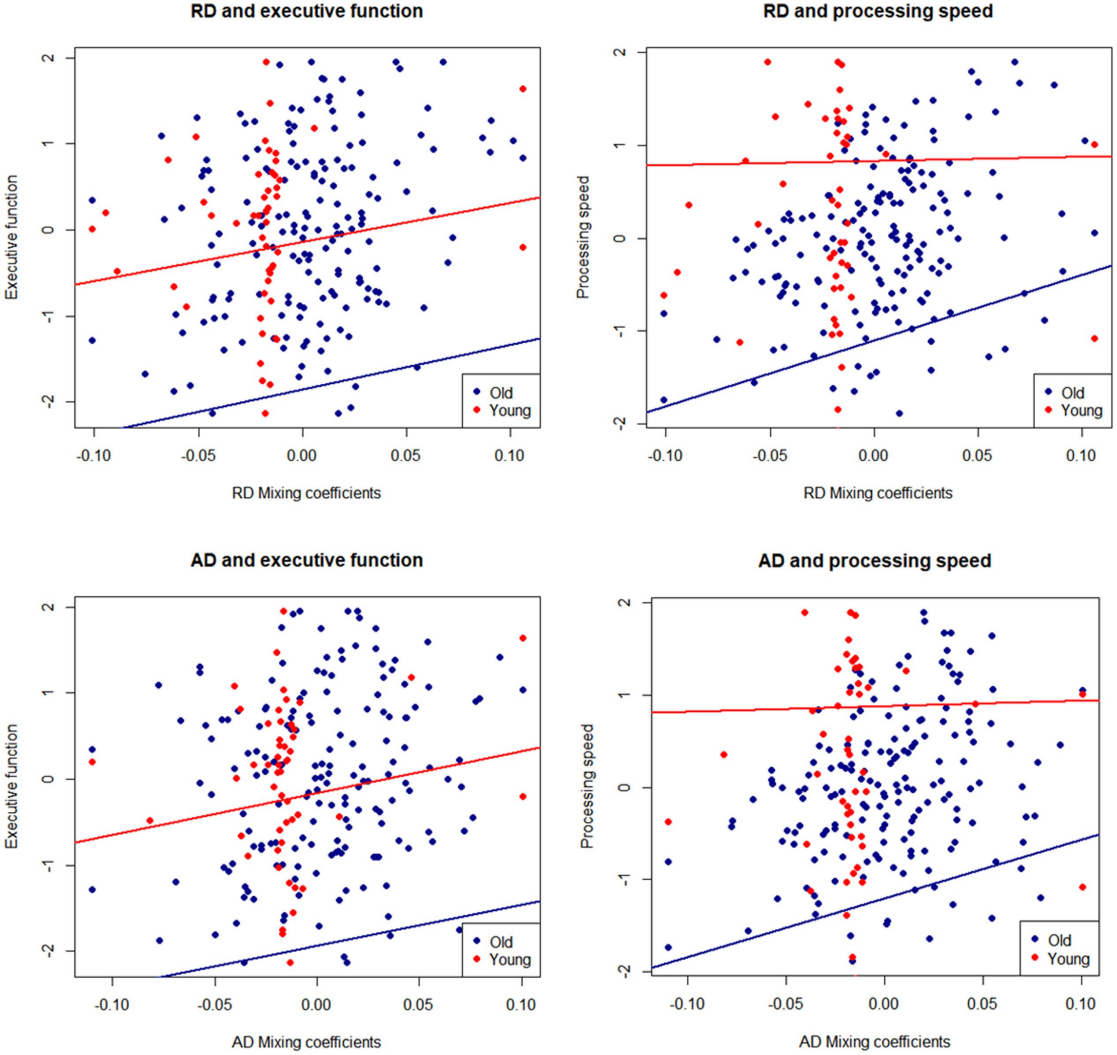
Mixing coefficients for IC2-RD and IC2-AD and association with executive function and speed composites. Lines of fit are adjusted by sex and education. Cognitive scores are residualized for age.

To test whether the age differences in RD and AD depicted by IC2 were relevant for cognition, we conducted regression analyses to examine the relationship between the mixing coefficients for RD and AD and the executive function and processing speed composites. Because both DTI values and cognition show strong associations with age, which may drive their correlation (11, 55), we residualized the executive function and processing speed controlling for age. Note that the mixing coefficients for RD and AD already contain age information, so they were not residualized. The scatterplots in Figure 3 display the relationship between the mixing coefficients and cognitive scores, while controlling for sex and education. The regression lines represent the results of the linear models fitted to the data. After controlling for these covariates and correcting for multiple comparisons, we found that higher mixing coefficients for RD and AD were associated with better executive functioning and processing speed.

To test whether the IC2-cognition association was present in both younger and older groups, we performed regression analyses by age group, adjusting for sex and education (Table 2). We found that the mixing coefficients for RD and AD were significant predictors of executive function and processing speed only among older adults but not among younger adults. In the older group, in addition to the mixing coefficients, education was a significant positive predictor of executive function and processing speed.

**Table 2:** Regression analyses of mixing coefficients for RD and AD as predictors of executive function and processing speed.

	Executive function						Processing speed					
	Young			Old			Young			Old		
	*β*	*p*	*q*	*β*	*p*	*q*	*β*	*p*	*q*	*β*	*p*	*q*
Model 1												
IC2-RD	0.110	0.442	0.530	0.186	0.010	0.004	0.009	0.921	0.980	0.321	0.001	0.003
Education	0.155	0.328	0.437	0.350	0.001	0.006	−0.271	0.013	0.026	0.272	0.001	0.003
Sex	0.760	0.202	0.404	−0.027	0.818	0.884	−0.010	0.980	0.980	0.154	0.091	0.156
Model 2												
IC2-AD	0.142	0.319	0.437	0.173	0.017	0.051	0.014	0.880	0.980	0.291	0.001	0.003
Education	0.155	0.139	0.333	0.363	0.001	0.006	−0.272	0.013	0.026	0.292	0.001	0.003
Sex	0.663	0.254	0.435	0.023	0.884	0.884	−0.010	0.972	0.980	−0.172	0.272	0.408

Table 2 displays the results of regression analyses examining the relationship between mixing coefficients for radial diffusivity (RD) and axial diffusivity (AD) and executive function and processing speed among young and old adults.

The fundamental question we were interested in answering is whether the multimodal fusion of DTI parameters using mCCA + ICA would provide more relevant information on age differences in WM concerning cognition than conventional, unimodal analysis. To investigate this, we conducted regression analyses between mean FA, MD, AD, and RD across the whole WM skeleton with executive function and perceptual speed scores, controlling for age, sex, and education. No association was significant after FDR correction. See Supplementary material 2 for more details.

## Discussion

We presented the first application of symmetric multimodal fusion analysis, mCCA + jICA, to characterize joint age differences in four DTI feature maps: FA, MD, AD, and RD, in only WM space. Our analyses revealed one high-stability modality-shared IC with co-variate patterns of RD and AD that differentiated between young and older adults. The joint information across RD and AD showed a superior association with cognitive performance compared to unimodal analyses.

### Joint differences in DTI parameters between young and older adults

In the context of our study, we can interpret the mixing coefficients as the strength of the covariance between the DTI features in expressing age differences in the WM microstructure for each IC. In other words, a higher mixing coefficient for RD and AD indicated stronger age differences in RD and AD in the regions indicated in IC-2. There are a couple of observations that we would like to highlight when interpreting mixing coefficients.

First, the variance in the mixing coefficients was greater in the old group than in the young group, consistent with age-related increases in heterogeneity, as previously described for other structural and functional brain features (56, 57). Second, we found more negative values of mixing coefficients in older participants, suggesting weaker associations between RD and AD within the IC2. It is possible that the negative mixing coefficients observed in older adults reflect a decrease in the spatial specificity of WM microstructures with age, in line with the dedifferentiation hypothesis, which posits that certain neural processes become less distinct and spatially specific with age (57). In this context, this could reflect an increased variability in the extent and localization of myelin loss or other histological processes. However, this possibility needs to be investigated by fusing features generated with MRI methods specific to myelin and axonal components such as myelin water fraction, neurite density orientation, and quantitative magnetization transfer (58–60). Additionally, it is worth noting that the results observed in the young group might be influenced by a restriction of range in the data, which could potentially affect the interpretation of the linear regression model results. Further investigation is needed to confirm and understand the implications of this limitation.

Overall, the results from the mCCA + jICA approach demonstrate a unique pattern of joint age differences in RD and AD. Modality-shared IC2 was localized to the splenium of the corpus callosum, internal capsule, and prefrontal WM. The genu of the corpus callosum is the primary late-myelinating WM region, achieving peak myelination ~70–109 weeks after birth (19). Related to this, it is characterized by small axon diameter, thin myelin sheaths, and a low oligodendrocyte-to-axon ratio, which makes its myelin sheaths metabolically challenged and more vulnerable to age-related deterioration (61). The splenium of the corpus callosum is also considered late-myelinating, with peak myelination achieved ~68 weeks after birth. The anterior internal capsule also has peak myelination achieved ~109 weeks after birth. In contrast, the posterior internal capsule is considered early-myelinating and begins myelinating <68 weeks before birth. Thus, our results support the retrogenesis pattern of WM degeneration, except for the voxels in the posterior internal capsule.

As known from unimodal analyses, age differences are typically characterized by decreased FA, increased MD and RD, and bidirectional differences in AD (11, 44, 62). In contrast, the mCCA+jICA showed no age differences in FA or MD, but rather a covariation of age bidirectional differences in RD and AD. However, the increases in RD were mostly localized to the genus of the corpus callosum, prefrontal WM, and anterior limb of the internal capsule, consistent with the retrogenesis hypothesis and vulnerability of myelin in late-myelinating regions.

We observed that increases in RD in the splenium of the corpus callosum and prefrontal/frontal WM were accompanied by lowered AD in the same regions. Studies using DTI-post-free water elimination have revealed that increases in RD accompany a decrease in AD with age, for example, in the frontal WM and parts of the corticospinal tracts (e.g., superior corona radiata) (63). Our earlier work also showed that increases in RD were accompanied by a decrease in AD in the superior corona radiata and prefrontal WM regions, but this effect was accompanied by decreased FA (11). Our study suggests that mCCA + jICA allows the detection of unique age differences driven by RD and AD independently of FA and MD.

In summary, mCCA+jICA is sensitive to the cross-information among all DTI features, which captures how DTI features interact and creates independent sources that explain unique mechanisms of WM aging (10). This multimodal fusion approach allowed us to revisit age differences in the entire WM using a data-driven approach. As hypothesized, this IC showed co-variant age differences in RD and AD in late-myelinating regions that may reflect demyelination, unrestricted diffusion of water–or chronic axonal loss (64, 65). Future studies should extend these results and test the utility of multimodal fusion using quantitative MR features with greater specificity for WM microstructure.

### Ability to detect age differences relevant to cognition

Associations of DTI with cognition (2) have been inconsistent, possibly due to multiple factors such as selective DTI parameter use, selective ROI, or type II error caused by multiple comparisons. We showed that mCCA + jICA could detect co-varying patterns of RD and AD that show a superior correlation with cognition than unimodal analyses, emphasizing the importance of studying WM MRI modalities together.

This first application of mCCA + jICA to study age differences in healthy aging WM identified multimodal patterns linked to executive function and processing speed composite scores. Specifically, RD-AD IC2 positively correlated with processing speed and executive function among the older adults, suggesting that RD and AD shared co-variance may capture a more nuanced pattern of age-related WM differences that correlates with cognition more robustly than any DTI feature alone.

The regression analyses indicated that education also had a positive effect on cognition among the older adults, which is consistent with the cognitive reserve theory (66). The fact that this positive effect was observed only in the older group may reflect a cumulative effect of past educational experiences, subsequent socioeconomic status, and environmental enrichment among older adults. In younger adults, this association may be more obscured given that the highest level of education determines peak cognitive performance and the age of maximal cognitive functioning (67), and that many of our younger participants were still continuing their education.

While our results showed a superior correlation with cognition compared to unimodal analyses, our multimodal fusion approach does not maximize both the inter-modality associations and the correlations with cognition. An extension of mCCA + jICA, mCCA + jICA with reference uses a supervised multimodal approach to maximize the correlation between cognitive scores and mixing coefficients (68). This supervised fusion approach can extract IC associated with a specific prior reference (e.g., cognitive scores) to optimize the decomposition of components and maximize the correlations with cognition. Future multimodal fusion studies should integrate mCCA + jICA and mCCA + jICA with reference to further study the patterns of WM aging, as well as the role of WM in key models of neurocognitive aging such as compensation (69), neural efficiency (70, 71), or dedifferentiation (57).

### Technical considerations and limitations

We need to consider several strengths and limitations in interpreting our results. First, we used the ICASSO algorithm to run multiple iterations of ICA and select the best single-run estimate to ensure the replicability of our results (46). This approach generates more reliable estimates for an IC than an estimate from a single run of the ICA algorithm (47). Since ICA algorithms (indeed most machine learning algorithms) are often stochastic in nature, replication requires addressing this aspect (72). Here we wanted to quantify the reliability of our ICA estimates to acquire more stable results. Currently, there are different strategies to evaluate the reliability of ICs using distinct clustering algorithms, including ICASSO. However, there are no current studies to establish the use of other measures of replicability/reliability of ICA results in DTI datasets, as most fusion models involve fMRI and EEG datasets (49, 73). Consequently, we chose a stricter quality index threshold from ICASSO to assess component stability. Future studies should explore using ICASSO and other clustering algorithms to estimate the stability of ICA components in DTI datasets.

Second, the four DTI parameters are based on the same diffusion tensor. These parameters can provide some unique information about tissue diffusivity; however, some microstructural processes in the WM present distinct patterns and combinations of increased/decreased FA, MD, RD, and AD (11). Thus, by fusing all four DTI parameter maps and maximizing the information from each DTI feature, we aimed to overcome—at least to some extent—the lack of specificity and mitigate the potential collinearity across the parameters. The mCCA + jICA model assumes some degree of correlation across modalities but allows accurate source separation based on the initial correlation between mixing profiles. In addition, mCCA + jICA has shown high accuracy in estimating independent sources, especially among sources derived from mixing profiles with distinct canonical correlation coefficients (74).

Another limitation is that DTI parameters reflect biological processes that depend on tissue architecture (e.g., in regions with crossing fibers). Because DTI confounds integrity, density, the diameter of myelin and axons, fiber orientational coherence, and the volume fraction of extracellular water (8, 75, 76), DTI alone may not be enough to study the aging WM. Future studies should attempt fusing modalities with greater sensitivity and specificity to myelin or axons, such as myelin water fraction, neurite density orientation, and quantitative magnetization transfer (58–60).

In addition, we used a model order of eight ICs, which is lower than the order of 12–15, typically used in mCCA + jICA analyses that include whole-brain data (48, 77). However, given that the WM skeleton occupies only ~8% of the total brain volume (137.832 skeleton voxels divided by 1.827.095 voxels of full-brain FA map in MNI space) in a sheath-like-structure and that structural data should exhibit fewer patterns that functional data, we concluded that eight ICs should provide enough flexibility in modeling age differences in WM. Although using the TBSS skeleton minimizes the effects of partial volume on DTI parameter values (78) in samples with a broad age span, it results in the data having a sheath-like structure, which may affect the component structure. We chose the TBSS approach for our study as it allows for representing local WM voxels and restricts the analyses to the center of WM tracts, reducing contribution from partial volume and white matter hyperintensities. Using skeletonized data at a 0.2 threshold also reduces the multiple comparisons problem and increases statistical power. While an ROI approach is typically preferred for confirmatory analyses, it would not be suited for mCCA + jICA which requires one continuous set of voxels for identifying patterns.

Lastly, because methods to estimate the number of components in data fusion have been developed using fMRI and EEG datasets (79), we estimated the number of components based on *a priori* knowledge of mechanisms of WM aging. As a result, we included the ICASSO algorithm in the mCCA + jICA framework to evaluate our components’ robustness and reliability carefully.

## Conclusion

Together, symmetric multimodal fusion (a) can provide new and potentially more rigorous information about brain aging, (b) can identify age differences in WM that bear more relevance to cognition than those obtained with traditional, region-based unimodal approaches. However, the DTI model, especially with a unimodal approach, provides limited information about the underlying neurobiological mechanisms of aging and dementia. Future multimodal fusion analyses should include more advanced MRI techniques sensitive to the WM’s microstructural tissue components and water-tissue interactions (80). Multimodal approaches allow leveraging the complementary information among different MRI modalities, representing an opportunity to characterize the role of WM connectivity in cognitive dysfunction and dementia.

## Data availability statement

The original contributions presented in the study are included in the article/Supplementary material, further inquiries can be directed to the corresponding author.

## Ethics statement

The studies involving human participants were reviewed and approved by the Institutional Review Board at the University of Illinois at Urbana-Champaign. The patients/participants provided their written informed consent to participate in this study.

## Author contributions

AMC and AZB contributed to the conception and design of the study and wrote the first draft of the manuscript. AMC organized the database and performed the statistical analysis. MH contributed to the statistical analysis. VDC contributed to the conceptualization of the study and statistical analysis. JF, ES, and NG contributed to data curation and data collection. EM and AFK contributed with the funding acquisition and project administration. All authors contributed to the article and approved the submitted version.

## Funding

The FAST sample data collection was supported by the National Institute on Aging at the National Institutes of Health (R37 AG025667) and funding from Abbott Nutrition through the Center for Nutrition, Learning, and Memory at the University of Illinois (PIs AFK and EZM). AZB (PI) and AMC’s work was supported by the Translational Medicine Institute Translational Acceleration Program at Colorado State University and the 1R21AG068939-01A1 from the National Institutes on Aging. VDC was supported in part by NIH R01MH118695 and NSF 2112455.

## Conflict of interest

The authors declare that the research was conducted in the absence of any commercial or financial relationships that could be construed as a potential conflict of interest.

## Publisher’s note

All claims expressed in this article are solely those of the authors and do not necessarily represent those of their affiliated organizations, or those of the publisher, the editors and the reviewers. Any product that may be evaluated in this article, or claim that may be made by its manufacturer, is not guaranteed or endorsed by the publisher.

## Footnotes

^1^ http://trendscenter.org/software/fit/

^2^ https://trendscenter.org/software/gift/
