# Supplementary material for: Symmetric data-driven fusion of diffusion tensor MRI: Age differences in white matter
Source: Front Neurol. 2023 Apr 17;14:1094313. doi: 10.3389/fneur.2023.1094313 (PMC10149813; doi:10.3389/fneur.2023.1094313)
Supplement: Supplementary file 2 [file Data_Sheet_2.docx]

**Supplementary Material 1**

Participant flow

For the older group, out of 213 participants who completed the clinical trial, 170 had good-quality DTI and T1-weighted data. Among the 43 excluded subjects, eight had insufficient brain coverage of b=0 images for T1w/T2w calculation, n=15 had missing DTI data due to technical problems, n=8 had anatomical abnormalities or ventriculomegaly, and n=12 had artifacts in DTI data. For the young sample with female dancers, 43 had good quality DTI and T1-W data, but 6 were excluded due to insufficient brain coverage of DTI data, resulting in 37 participants being included. All 14 participants had good quality DTI and T1-W data for the young sample of college-age adults. Our final sample comprised 170 older adults (aged 60-80) and 51 younger adults (aged 18-33).

**Supplementary Material 2**

Multiple linear regression models between DTI parameters and cognitive composites

|  | Executive Function | | | Processing Speed | | |
| --- | --- | --- | --- | --- | --- | --- |
|  | *β* | *p* | *q* | *β* | *p* | *q* |
| **Model 1** |  |  |  |  |  |  |
| RD | 0.143 | 0.495 | 0.720 | -0.123 | 0.343 | 0.449 |
| Age (years) | -2.126 | 0.001 | 0.004 | -1.833 | 0.001 | 0.004 |
| Education (years) | 0.740 | 0.009 | 0.020 | -0.165 | 0.197 | 0.364 |
| Sex | 0.154 | 0.816 | 0.956 | -0.271 | 0.374 | 0.449 |
|  |  |  |  |  |  |  |
| **Model 2** |  |  |  |  |  |  |
| AD | 0.056 | 0.890 | 0.956 | -0.276 | 0.122 | 0.364 |
| Age (years) | -2.161 | 0.001 | 0.004 | -2.038 | 0.001 | 0.004 |
| Education (years) | 0.739 | 0.007 | 0.020 | -0.158 | 0.214 | 0.364 |
| Sex | 0.211 | 0.751 | 0.956 | -0.257 | 0.393 | 0.449 |
|  |  |  |  |  |  |  |
| **Model 3** |  |  |  |  |  |  |
| FA | 0.631 | 0.069 | 0.110 | -0.028 | 0.853 | 0.853 |
| Age (years) | -1.787 | 0.001 | 0.004 | -1.816 | 0.001 | 0.004 |
| Education (years) | 0.727 | 0.012 | 0.024 | -0.161 | 0.210 | 0.364 |
| Sex | 0.036 | 0.956 | 0.956 | -0.314 | 0.303 | 0.440 |
|  |  |  |  |  |  |  |
| **Model 4** |  |  |  |  |  |  |
| MD | -0.592 | 0.046 | 0.081 | -0.099 | 0.450 | 0.480 |
| Age (years) | -1.973 | 0.001 | 0.004 | -1.825 | 0.001 | 0.004 |
| Education (years) | 0.723 | 0.008 | 0.020 | -0.158 | 0.216 | 0.364 |
| Sex | -0.050 | 0.940 | 0.956 | -0.371 | 0.228 | 0.364 |

Sex is coded as 0=female, 1=male. β represents the standardized coefficients, *p* represents the uncorrected p-value, and *q* represents the false discovery rate corrected p-value. Model 1 includes RD in the whole white matter, education (years), and sex. Model 2 includes AD in the whole white matter, education (years), and sex. Model 3 includes FA in the whole white matter, education (years), and sex. Model 4 includes MD in the whole white matter, education (years), and sex.
